# A Presentation of Fungating Nevoid Basal Cell Carcinoma Syndrome

**DOI:** 10.7759/cureus.98759

**Published:** 2025-12-08

**Authors:** Oluwafunke O Ogunremi, Brant Hannahs, Kirstin Hockhausen

**Affiliations:** 1 Department of Internal Medicine, University of South Dakota Sanford School of Medicine, Rapid City, USA; 2 Department of Dermatology, University of South Dakota Sanford School of Medicine, Rapid City, USA

**Keywords:** basal cell carcinoma variants, basal cell nevus syndrome, bcns, gorlin syndrome, nbccs, nevoid basal cell carcinoma syndrome

## Abstract

Gorlin syndrome, also known as nevoid basal cell carcinoma syndrome (NBCCS), is a rare autosomal dominant disease marked by the development of numerous basal cell carcinomas (BCC) that tend to occur at young ages. Here the authors detail a case of a young adult Caucasian female with both a complex social and medical history. These social determinants of health played a significant role during her management and treatment plan. Although the patient had many eruptions of BCC throughout her body, one chronic, non-healing BCC lesion located amidst a shoulder wound would eventually turn into an ulcerative fungating mass, resulting in amputation of the left arm with recurrence of the BCC at the site of amputation. The patient’s lesions were targeted with a combination of electrodessication and curettage (ED&C), Mohs surgery, and cemiplimab immunotherapy.

## Introduction

Gorlin syndrome, also known as nevoid basal cell carcinoma syndrome (NBCCS), is a rare autosomal dominant disease marked by the development of numerous basal cell carcinomas (BCCs) that tends to occur between puberty and age 35 [1]. In the United States, the prevalence of Gorlin syndrome is one in 31,000, with an equal male-to-female ratio [2]. This condition arises from mutations that disrupt the Hedgehog signaling pathway, most frequently involving the patched 1 (PTCH1), patched 2 (PTCH2), and suppressor of fused homolog (SUFU) genes [3]. These mutations result in constitutive activation of the pathway, promoting unchecked proliferation and tumor growth [4].

We report a case of Gorlin syndrome in a middle-aged adult female patient, whose diagnosis was established following extensive workup. This case highlights the importance of a multidisciplinary approach and a high index of suspicion when evaluating patients with compatible clinical features.

## Case presentation

A diagnosis of NBCCS can be established based on any of the following: (1) one major criterion with molecular confirmation; (2) two major criteria; or (3) one major and two minor criteria (Tables 1, 2) [5].

**Table 1:** Major Diagnostic Criteria of NBCCS and Patient Status Major diagnostic criteria for NBCCS and diagnosis is made based on the presence or absence of these criteria in the patient. *Right palmar and plantar pits were present in the subject patient. NBCCS: Nevoid basal cell carcinoma syndrome

Major Criteria	Present or Absent
One basal cell carcinoma (BCC) under the age of 20 years	Unknown
Multiple BCCs	Present
Odontogenic keratocyst of the jaw proven by histology in an individual younger than 20 years	Absent
Two or more palmar or plantar pits	Present*
Lamellar (sheet-like) calcification of the falx cerebri or clear evidence of calcification in an individual younger than age of 20 years	Absent
Childhood medulloblastoma	Absent
First-degree relative with NBCCS	Absent

**Table 2:** Minor Diagnostic Criteria of NBCCS and Patient Status Minor diagnostic criteria for NBCCS is presented and diagnosis is made based on the presence or absence of these criteria in the subject patient. *Mild straightening of the cervical and lumbar lordosis and thoracic kyphosis was present in the subject patient. NBCCS: Nevoid basal cell carcinoma syndrome

Minor Criteria	Patient Status
Rib anomalies (i.e., bifid, fused, or markedly splayed ribs)	Absent
Macrocephaly	Absent
Cleft lip or palate	Absent
Other specific skeletal abnormalities	Present*
Lymphomesenteric cysts	Absent
Ovarian or cardiac fibroma	Absent
Ocular anomalies (i.e., strabismus, hypertelorism, congenital cataracts, glaucoma, coloboma)	Absent

Evaluation, diagnosis, and treatment

**Figure 1:**
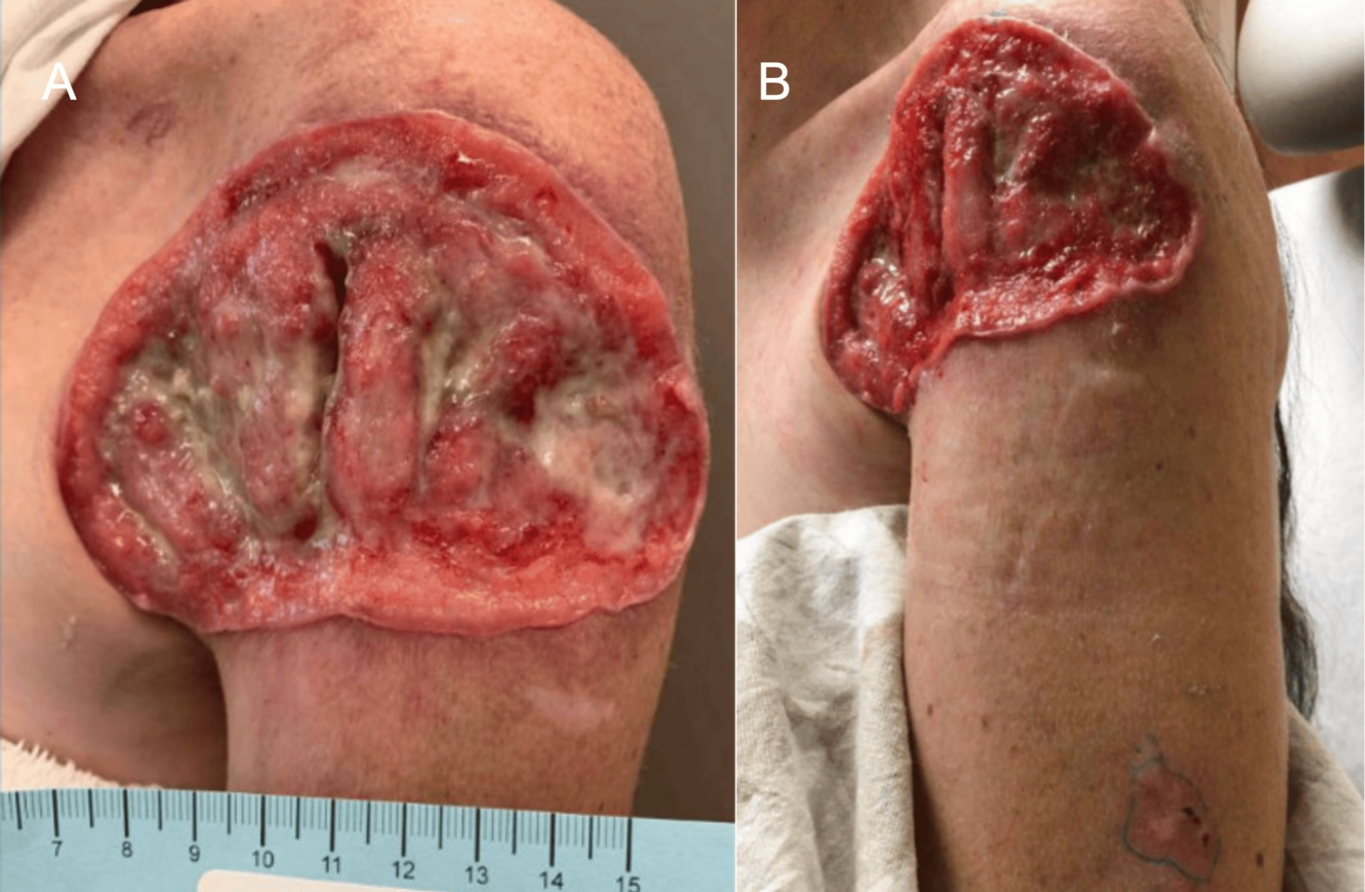
Left Shoulder Wound Presentation. Part 1. Initial clinical photographs of the patient's wound and skin presentation during an initial emergency department encounter. (A) Depicts a large full thickness ulceration on the left anterior shoulder with purulent drainage from a close range view. (B) Depicts a large full thickness ulceration on the left anterior shoulder with purulent drainage from a wide range view.

The subject in this investigation is a 39-year-old female patient who initially presented to the emergency department in December 2021 at age 36 for the evaluation of a chronic left shoulder wound. This patient’s medical history is significant for BCCs, malnutrition, dermatitis, Hepatitis C, anemia, heroin abuse, and methamphetamine abuse. At this visit, the patient informed the providers that the wound on her left shoulder had been present for one to two years but was unsure as to when and how it had initially begun. One year prior, the patient had a biopsy at that site for evaluation of BCC in another state. Following this, the patient was involved in a motor vehicle collision in which her seatbelt caused wound dehiscence. She reported that a “smelly” drainage at the wound site for the past two months prompted her to seek further care at this time. The patient also stated that a radiating pain to the left midsternal chest from the wound site on the left shoulder has been occurring. Physical examination at that time showed a large open granulating wound on the left anterior shoulder with purulent drainage (Figure 1). Probing the wound did not display any tunneling areas. There were no signs of erythema extending beyond the wound borders or induration. Wound culture was collected and grew moderate gram-positive and gram-negative organisms (Table 3). Sulfamethoxazole-trimethoprim (Bactrim) was prescribed to address the infection.

**Table 3:** Wound Culture Results The results from wound cultures obtained from the patient’s left anterior shoulder during an emergency department visit are summarized.

Wound Culture Results	Extensivity
Staphylococcus aureus	Several (<40%)
*Streptococcus dysgalactiae *subsp.* equisimilis*	Many (40-60%)
Moraxella catarrhalis	Several (<40%)
*Corynebacterium striatum *group	Several (<40%)
Fusobacterium nucleatum	Several (<40%)
Bacteroides fragilis	Many (>50 colonies)
Peptostreptococcus anaerobius	Many (>50 colonies)

**Figure 2:**
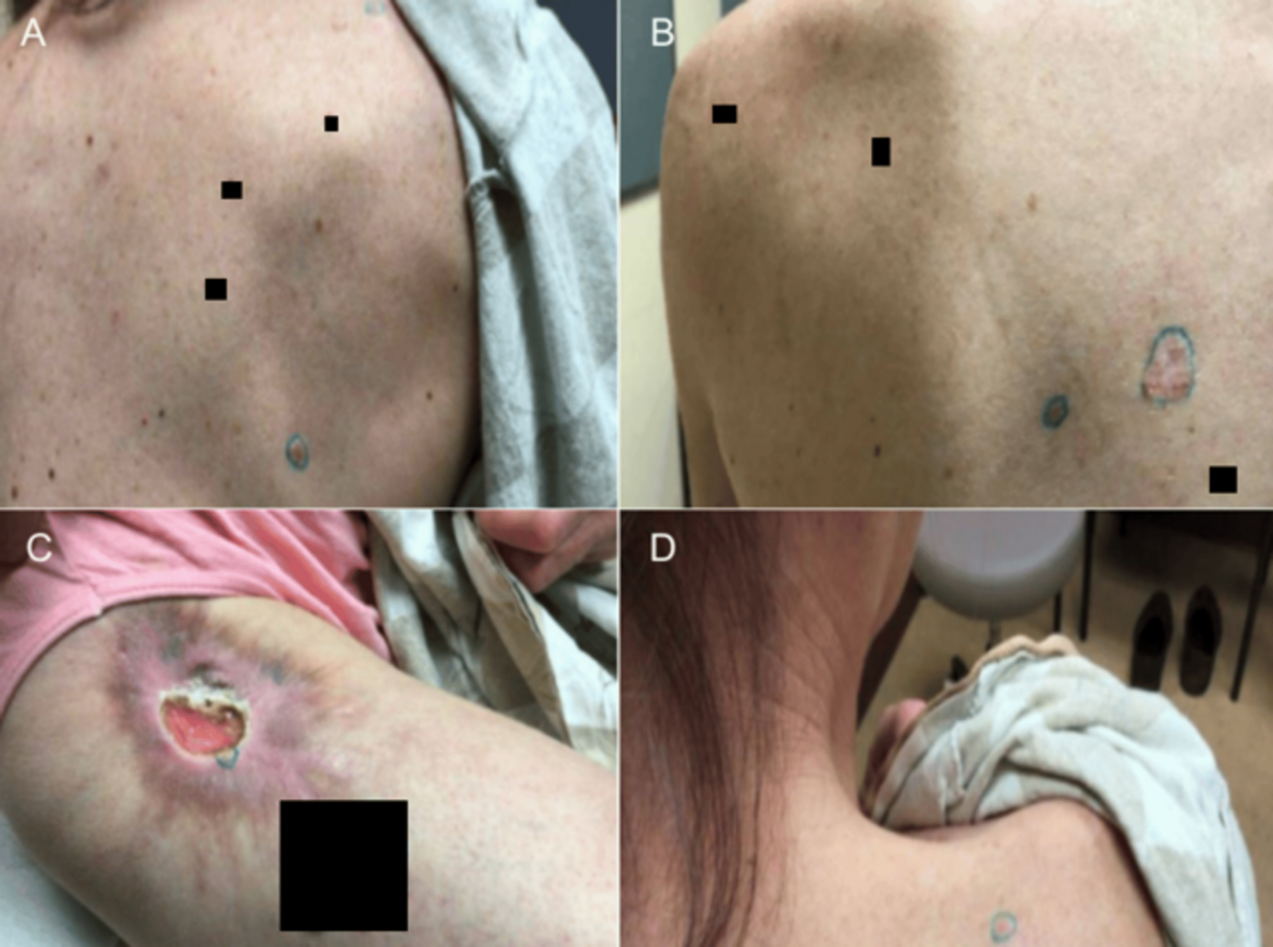
Initial Outpatient Dermatology Presentation. Part 1 Locations of the biopsy sites at an outpatient dermatology visit. (A) Biopsy site pictured on the right scapular back demonstrating a pink shiny papule. (B) Biopsy sites pictured on the left of midline interscapular back and left inferior scapular ridge, respectively, demonstrating a pink shiny papule and a scaly white to pink plaque. (C) Biopsy site pictured on the right proximal anterior thigh demonstrating a pink plaque with central ulceration and white rim surrounded by a hyper-pigmented, irregular border. (D) Biopsy site pictured on the right posterior superior shoulder demonstrating a pink shiny papule.

**Figure 3:**
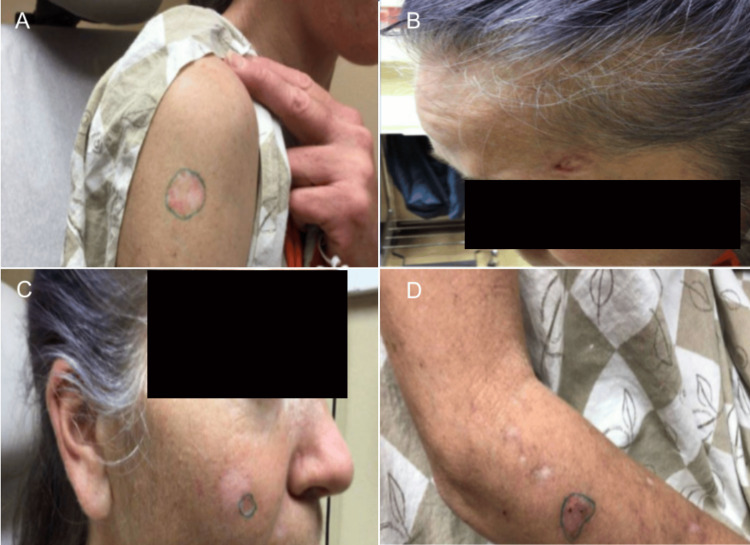
Initial Outpatient Dermatology Presentation. Part 2 Locations of the biopsy sites at an outpatient dermatology visit. (A) Biopsy site of a white and pink plaque on the right anterior deltoid. (B) Biopsy site of a pink pearly papule with a central brown macule on the left temple. (C) Biopsy site of a white plaque with an irregular pink border on the right mid cheek. (D) Biopsy site of a pink plaque with speckled medium brown pigmentation on the right proximal dorsal forearm.

Two weeks after the encounter at the emergency department, the patient attended an outpatient dermatology visit. At this visit, the patient recounted having a scar like bump on the left shoulder even as a child. She confirmed that she developed a big sore on this same shoulder that had been present for a minimum of 18 months (Figure 1). The patient stated that this spot has been previously biopsied by a different provider and was found to be BCC. An additional biopsy was performed at that time and was also found to be BCC; however, previous medical records were unable to be obtained. At the time of this current visit, several biopsies throughout the body were taken and identified to be BCCs (Figures 2, 3 and Table 4). The patient was informed of the diagnoses but unfortunately did not follow-up and was unable to be reached on her line by the dermatology medical team.

**Figure 4:**
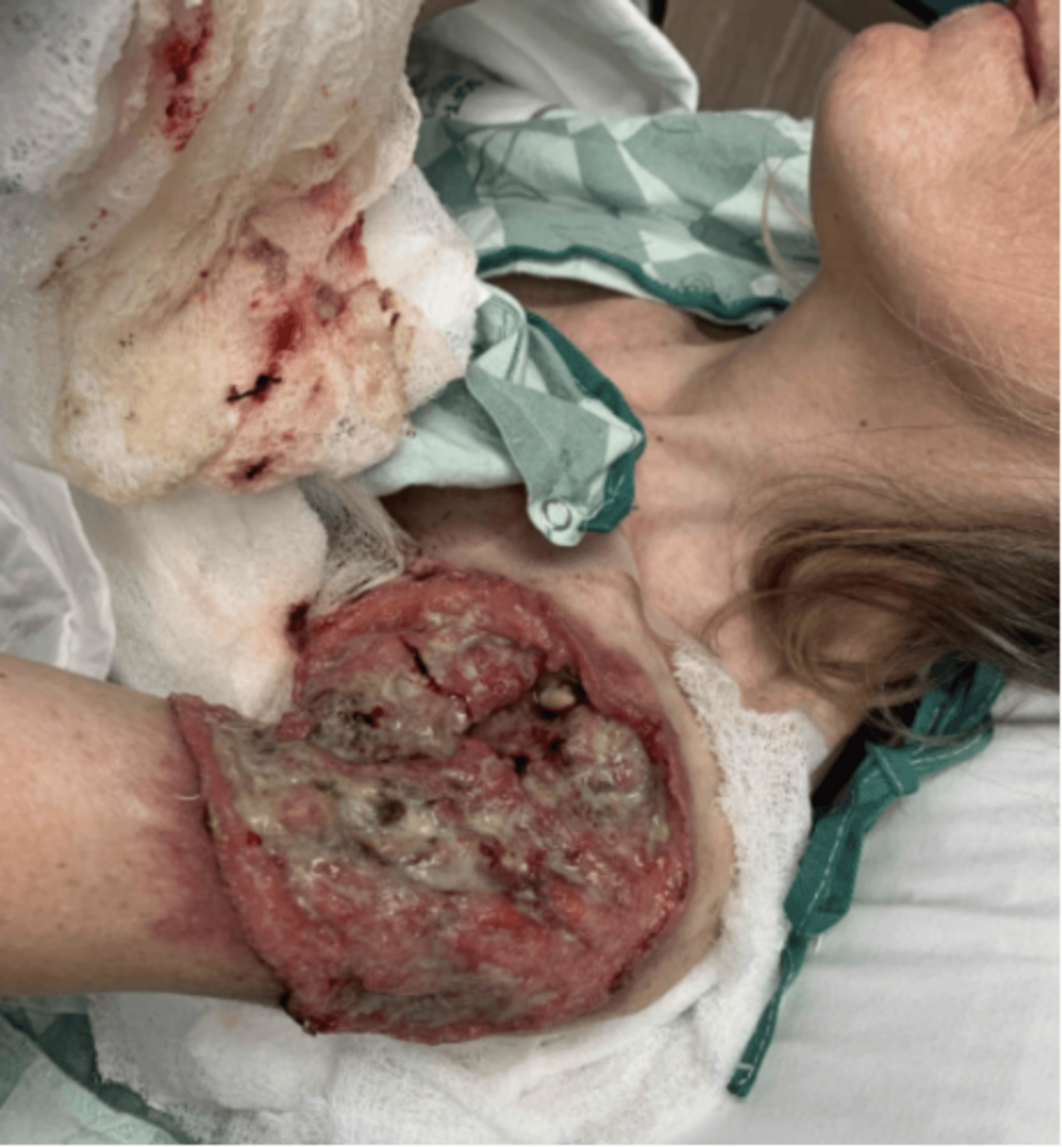
Left Shoulder Wound Presentation. Part 2. A large ulcerating wound with purulent drainage present on the left anterior shoulder as seen in the emergency department visit.

The patient was temporarily lost to follow-up but was later seen again in the emergency department in February 2023. During this encounter, she presented with an increasingly painful and enlarging wound of the left shoulder. Physical examination noted a large ulcerating wound of the anterior left shoulder with malodorous purulent drainage (Figure 4). Treatment with antibiotics, IV fluids, and pain management was begun. CT imaging of the left shoulder at this time showed enlarged lymph nodes, concerning for malignant metastasis. The patient was deemed to have capacity and voiced understanding but chose to leave the facility against medical advice.

**Figure 5:**
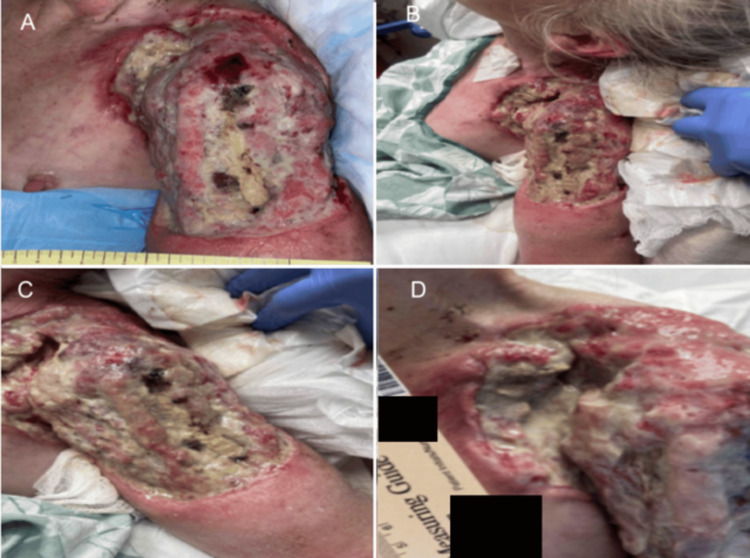
Left Shoulder Wound Presentation. Part 3. (A-D) A large, purulent, fungating wound present over the patient's left shoulder, axillae, and scapulae in different views.

**Figure 6:**
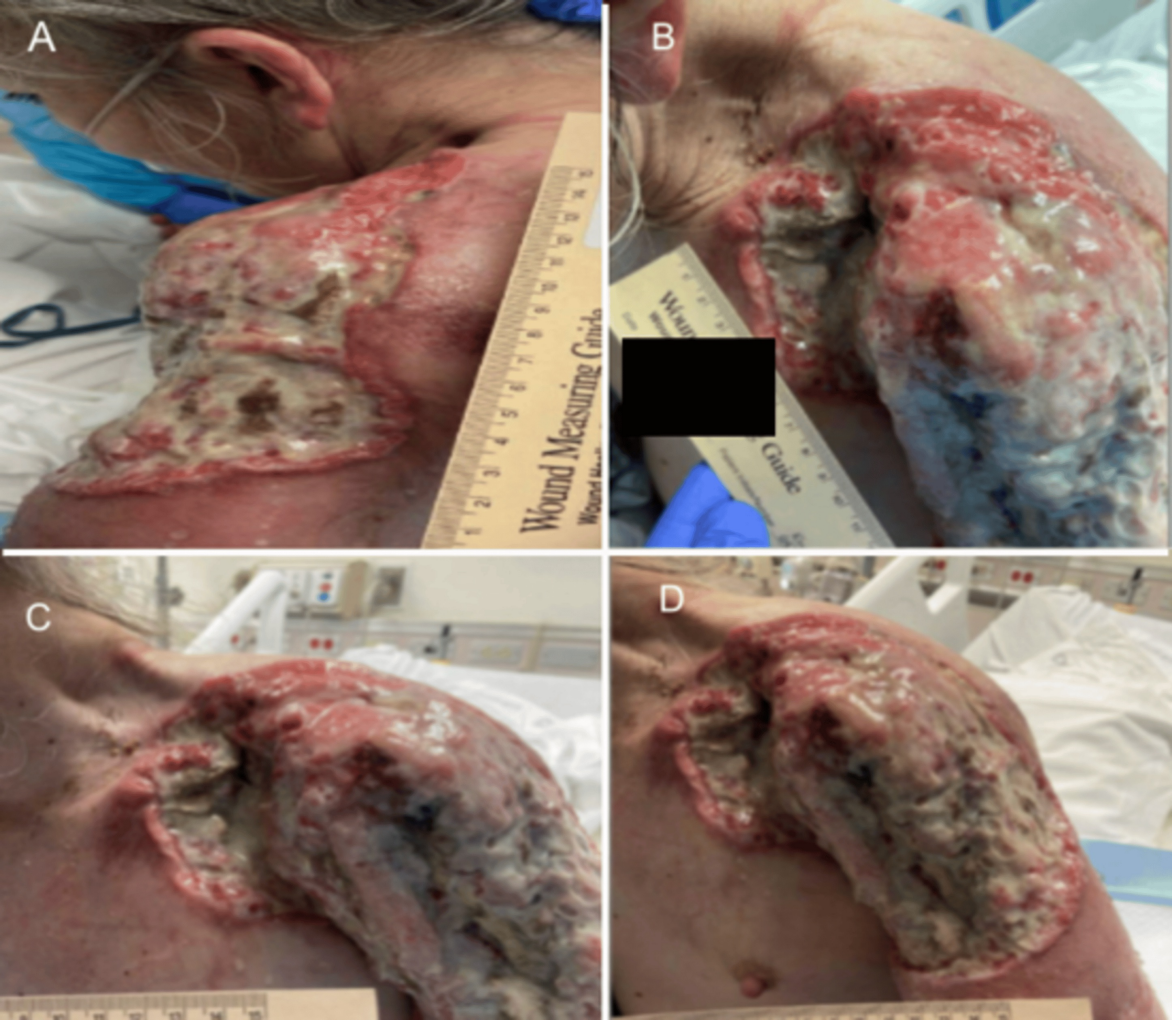
Left Shoulder Wound Presentation. Part 4. (A-D) Additional images of a large, purulent, fungating wound present over the patient's left shoulder, axillae, and scapulae in different views.

**Figure 7:**
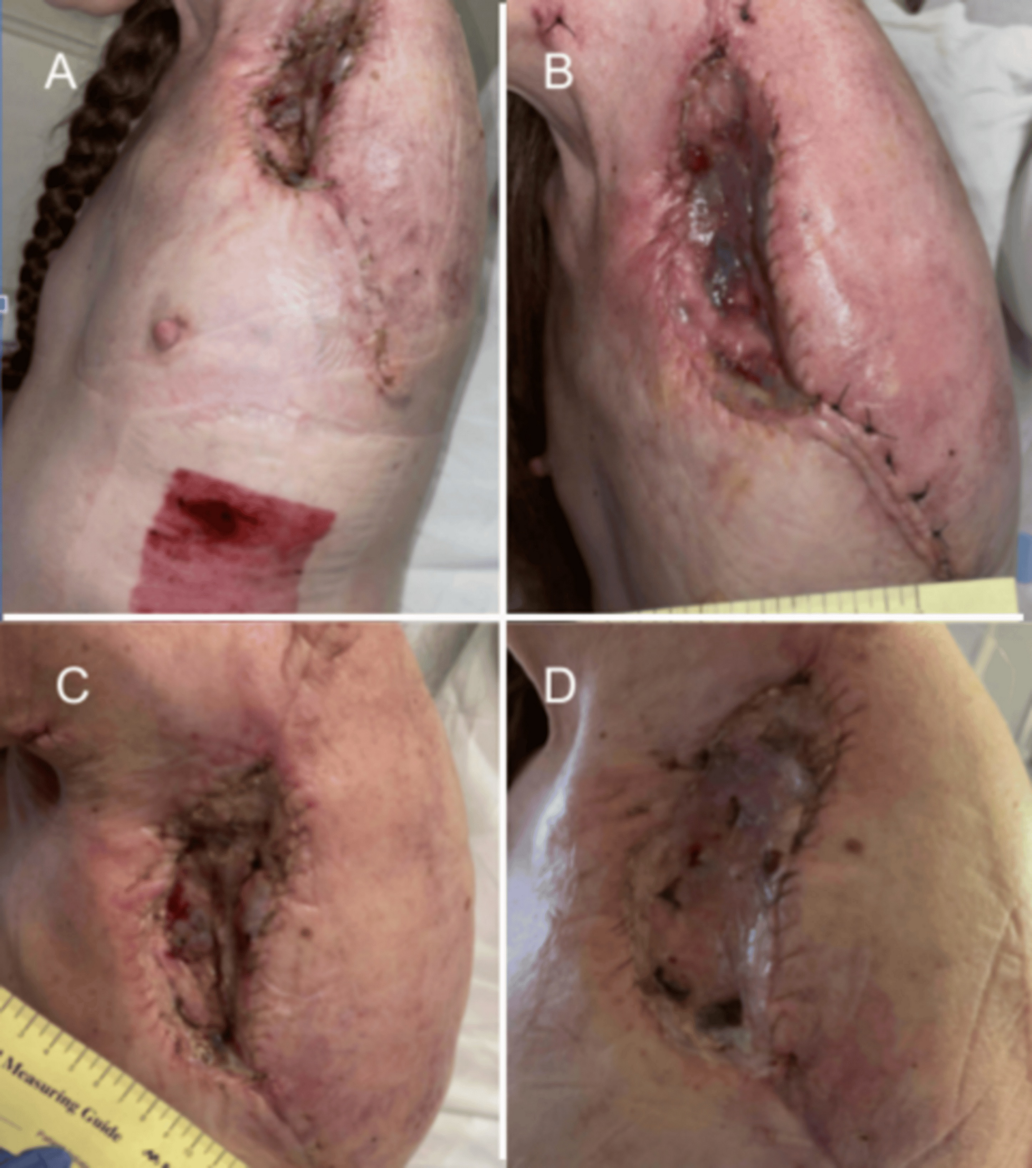
Left Shoulder Wound Presentation. Part 5. (A-D) Images of the patient’s left shoulder at the site of surgical amputation from multiple views. Well healing surgical incisions, surrounded by faint erythema can be seen.

In November 2023, the patient returned to the emergency department and fungating BCC of her left shoulder axillae and scapulae was examined (Figures 5, 6). Treatment with IV fluids, pain management, and antibiotics was begun. A wound debridement and lavage procedure was performed by general surgery. Days later, plastic surgery was performed for deep muscle and lymph node biopsies of the perimeter of the left shoulder axilla and arm wound. Biopsy results showed that left-sided supraclavicular, axillary and pectoral lymph nodes were negative for metastasis. Approximately a month later, a left forequarter amputation with neuroplasties of the brachial plexus lateral cord, medial cord and posterior cord with placement of an implantable pain pump was performed (Figure 7). A few days following this procedure, a left chest split thickness skin graft was performed. Over the following months, the site of amputation healed appropriately. In January 2024, the patient was seen by oncology following hospital discharge. The patient was recommended to complete PET/CT and then begin targeted therapy with vismodegib. PET/CT at this time demonstrated residual disease in the surgical area. She took vismodegib for a short time before discontinuing it due to hair loss, fatigue, and muscle pain. Unfortunately, the patient canceled follow-up visits and was last to follow-up until six months later.

**Figure 8:**
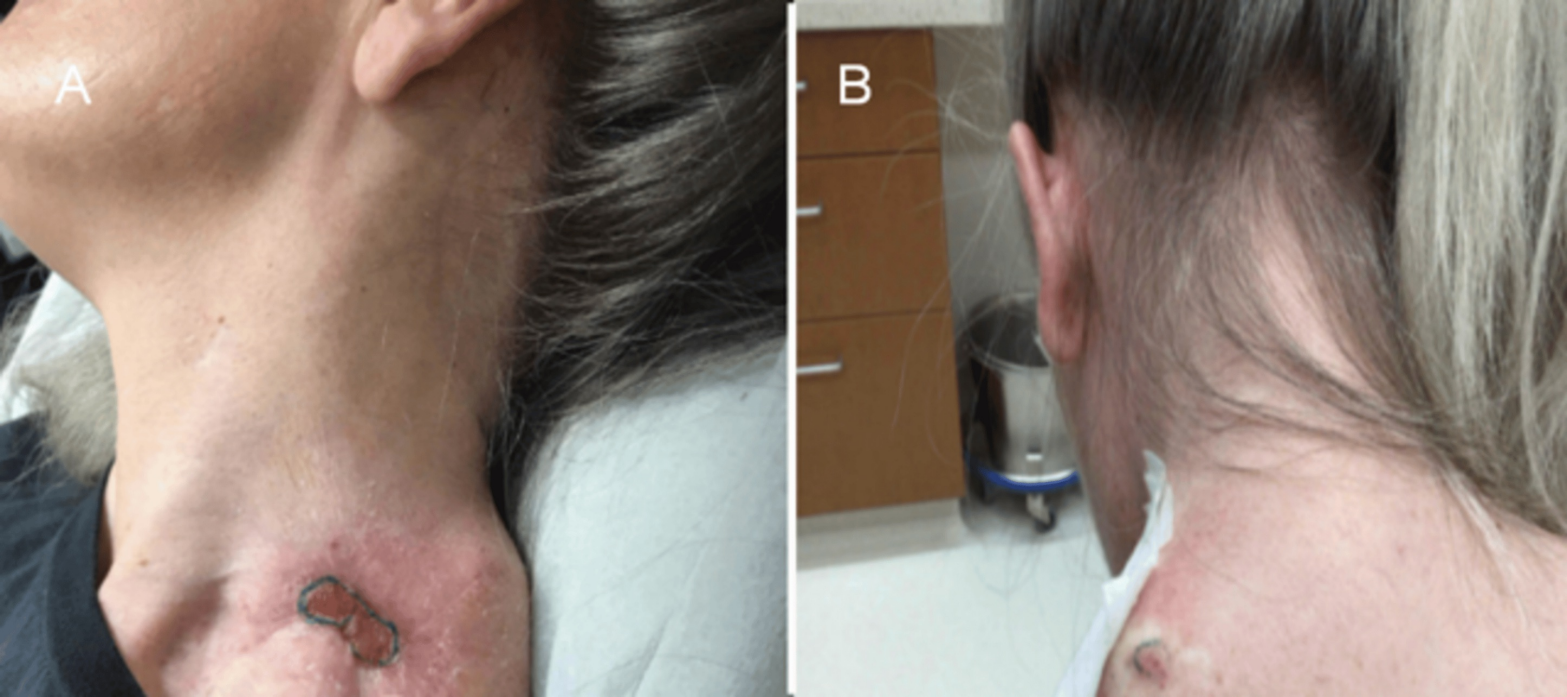
Left Shoulder Wound Presentation. Part 6. The focal area of concern for the re-occurrence of BCC.  (A,B) Located on the left shoulder at the prior site of amputation there is a red, scaly plaque with irregular borders. BCC: Basal cell carcinoma.

**Figure 9:**
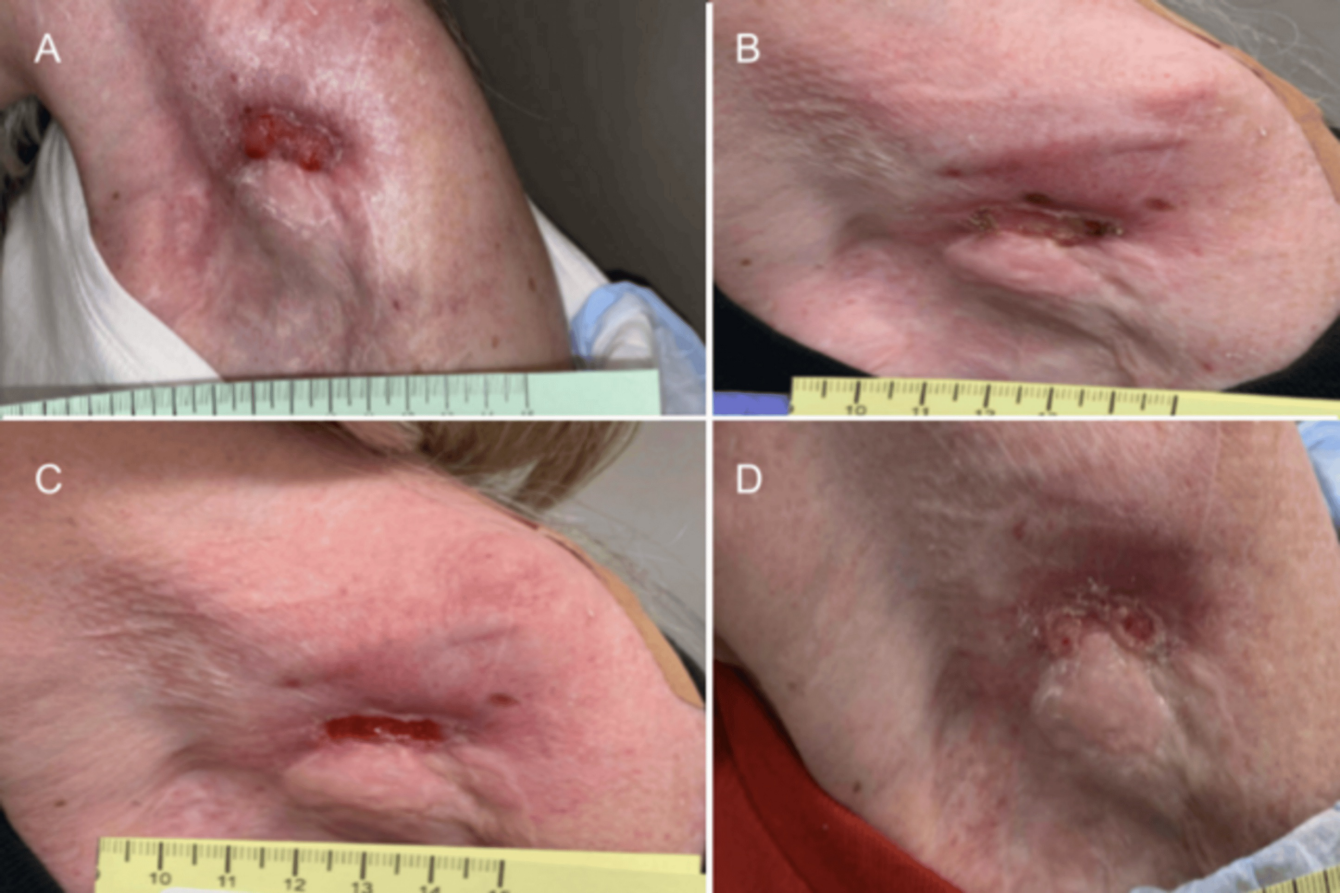
Left Shoulder Wound Presentation. Part 7. (A-D) Further progression of the lesion at the site of amputation on the left shoulder, concerning for reoccurrence of BCC. Pictured is a red, scaly plaque with central erosion on the left shoulder at different stages of progression, monitored over several clinic visits. BCC: Basal cell carcinoma.

In July 2024, the patient reestablished and completed a PET/CT, which demonstrated hypermetabolic activity and increased size in the soft tissue surrounding the left shoulder surgical site, right inguinal lymphadenopathy, new skin nodularity in the right lower leg, elevated activity in the soft tissue of the right anterior lateral hip, and hypermetabolic activity in the mediastinum. The right-sided lymphadenopathy and right lower leg nodularity were favored to be reactive due to cellulitis that had previously occurred in this limb. The patient was advised and agreed to start immunotherapy with cemiplimab at this time. At this point, the patient was diagnosed as having stage four BCC. The patient was again lost to follow-up until January 2025. In January 2025, the patient began cemiplimab. In February 2025, PET/CT demonstrated vascular disease, increased size and activity in the soft tissue of the left shoulder in addition to increased activity in the right inguinal and external iliac lymph nodes. At this time, the patient was seen by outpatient dermatology. A full-body skin check was performed, and a surgical plan was set in place for the previously identified skin cancers (Table 4). The patient was scheduled for the following procedures: Mohs surgeries and destruction of malignant lesions via electrodessication and curettage (ED&C). In March 2025, the patient returned for successful management and treatment of these lesions over a series of several visits. In late March, it was deemed that a discrete area at the amputation site of the left upper arm was suspicious for a non-melanoma skin cancer (Figures 8, 9). A biopsy was taken, and the results indicated a recurrent BCC.

**Table 4:** Skin Biopsy Results Summary of the locations, results, and management of the various skin biopsies performed on the patient. *N/A: A surgical plan was not needed for the sites on the left anterior shoulder and left mid upper arm due to the amputation of the left arm. ED&C: Electrodessication and curettage; BCC: basal cell carcinomas.

Biopsy Location	Features	Diagnosis	Surgical Plan
Right Mid Cheek	Superficial, Nodular and Infiltrative Patterns	BCC	1.4 cm Mohs
Left Medial Frontal Hairline	Nodular Pattern	BCC	6 mm Mohs
Right Proximal Dorsal Forearm	Nodular Pattern	BCC	2.0 cm ED&C
Right Anterior Deltoid	Superficial and Nodular Patterns	BCC	3.2 cm ED&C
Right Scapular Back	Nodular and Micronodular Patterns	BCC	1.1 cm ED&C
Left of Midline Interscapular Back	Nodular Pattern	BCC	2.8 cm Mohs
Right Proximal Anterior Thigh	Dermal Fibrosis and Epidermal Hyperplasia with Mild Cytologic Atypia	Nonspecific Findings	11 cm - re biopsy (punch)
Left Inferior Scapular Ridge	Superficial and Nodular Patterns	BCC	6 mm ED&C
Right Posterior Superior Shoulder	Superficial and Nodular Patterns	BCC	6 mm ED&C
Left Anterior Shoulder	Nodular and Infiltrative Patterns	BCC	*N/A*
Left Temple	Nodular Pattern	BCC	2 cm Mohs
Left Mid Upper Arm	Nodular and Infiltrative Patterns	BCC	*N/A*

Diagnostic workup and family history

In February 2025, when the patient reestablished with outpatient dermatology, a thorough workup was conducted to assess for the presence of other minor and major criteria for NBCCS. X-rays of the right hand with three views were obtained to evaluate for lytic bone lesions. No abnormalities were visualized via X-ray. X-rays of the skull with four views were obtained to evaluate for calcified falx cerebri, bridging of sella turcica, odontogenic keratocysts of the jaw, and broad nasal root. No abnormalities were visualized via X-ray. The patient reported a history of skin cancer in her father and paternal grandfather but was unsure of their definitive diagnoses.

## Discussion

**Figure 10:**
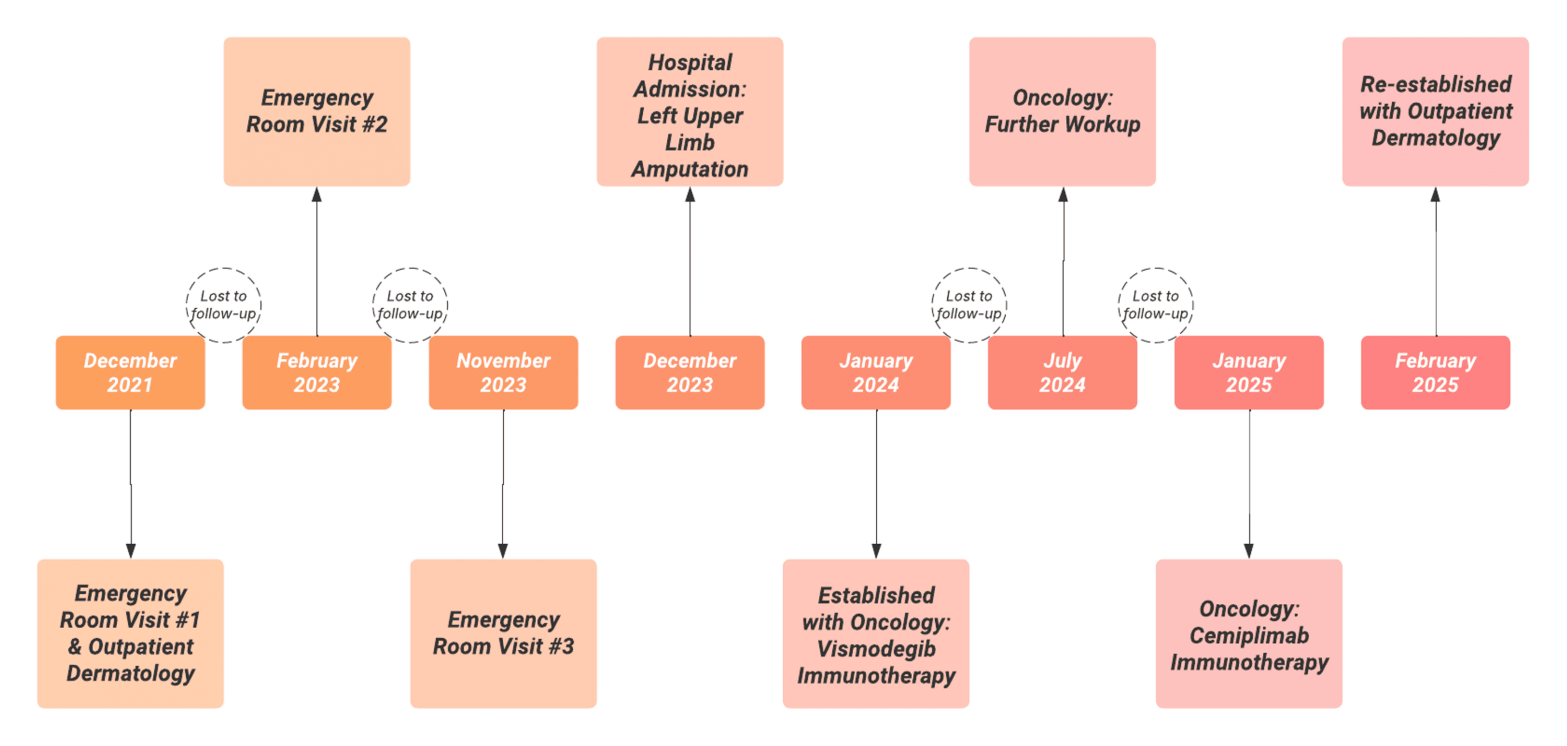
Medical Treatment Timeline Timeline of the subject patient’s medical visits. The patient was lost to follow-up several times due to the social determinants of health.

This case highlights a classic diagnosis of NBCCS, or Gorlin syndrome, in a 39-year-old female patient who initially presented to the emergency department with a chronic, non-healing shoulder wound. The protracted course of this lesion, present for one to two years with a history of prior BCCs at the same site, along with the emergence of multiple new BCCs, underscores the importance of recognizing NBCCS in patients with recurring or early-onset BCCs, particularly when located in non-sun-exposed areas. This patient was lost to follow-up several times, which further complicated the identification of the reoccurrence of the disease and administration of appropriate medical treatment (Figure 10).

NBCCS is a rare autosomal dominant disorder characterized by a constellation of findings, including multiple BCCs, odontogenic keratocysts, palmar or plantar pits, and skeletal abnormalities [6]. While BCCs are most commonly associated with UV exposure, the presence of numerous or recurrent BCCs in atypical locations, such as the shoulder in this case, should prompt consideration of a syndromic etiology [7]. The patient in this case recalled having a lesion on her shoulder since childhood, suggesting the potential presence of a long-standing BCC, which can be seen in cases of NBCCS as patients may begin developing BCCs at a young age [1].

The treatment for this condition varies depending on the clinical presentation. Management can be difficult, as there is a lifelong risk for the development of new lesions or tumors. For BCCs, there are numerous options for treatment. The primary goal for BCC treatment is the removal of the lesion. Curettage and electrodessication are highly effective ways to remove small lesions, while cryotherapy, Imiquimod cream, and surgical excision can be tools used for removal as well [8]. For large, aggressive, or high-risk lesions, Mohs micrographic surgery remains the gold standard due to its tissue-sparing and high cure rate properties [9]. A promising new treatment emerging for BCCs, especially those in NBCCS, is vismodegib and sonidegib, which function as a hedgehog pathway inhibitor [10,11]. The number and severity of BCCs and other manifestations are influenced by both genetic and environmental modifiers, with no consistent genotype-phenotype correlation established [12,13]. However, patients with skeletal anomalies are at higher risk for severe disease, including increased BCC burden and other neoplasms [14]. Patients should be counseled to adhere to strict photoprotective measures in order to reduce the risk of BCC recurrence [15]. Skeletal anomalies like bifid ribs, wedge-shaped vertebrae, and scoliosis can occur and referral to orthopedic specialists for assessment and management may be indicated [7]. For keratocystic odontogenic tumors, they can be treated with enucleation, and following that with chemical or mechanical curettage decreases recurrence [8]. Regular dental surveillance with orthopantograms every 12-18 months from age eight is recommended [7]. Dentists often play a crucial role in early identification due to the frequent occurrence of jaw cysts [16,17]. Surveillance brain MRI is indicated in early childhood, especially for those with SUFU mutations, due to increased medulloblastoma risk [18]. Genetic counseling is recommended for affected individuals and their families to guide surveillance and inform risk of associated complications [16].

This case reinforces the importance of a thorough dermatologic and family history, especially in patients with recurrent BCCs or longstanding cutaneous lesions. Although genetic testing was negative in this case, the clinical picture strongly supports a diagnosis of NBCCS based on major and minor diagnostic criteria. Early diagnosis is crucial, as it can help minimize the severity of complications such as malignant skin and brain tumors, as well as prevent extensive jaw cyst destruction and secondary oral-maxillofacial deformities [19,20].

## Conclusions

A complicated case of NBCCS is presented in this article. The patient had diffuse BCC lesions present throughout her body with a recurrent lesion over a site of previous limb amputation. This patient’s treatment regimen consisted of a combination of ED&C, Mohs surgery, and cemiplimab immunotherapy. Although not discussed to conceal patient identity, many adverse social determinants of health directly affected this patient, leading to the outcome reported here. Late diagnosis in adulthood, in conjunction with unstable living conditions, leading to the patient being lost to follow-up several times, greatly contributed to delayed management and rapid progression of the disease courses. The clinical picture described serves as an important reminder to both patients and physicians of the importance of regular health maintenance appointments and reducing barriers to healthcare access.
